# Dependence on Film Thickness of Guest-Induced *c* Perpendicular Orientation in PPO Films

**DOI:** 10.3390/polym13244384

**Published:** 2021-12-14

**Authors:** Baku Nagendra, Emanuele Vignola, Christophe Daniel, Paola Rizzo, Gaetano Guerra

**Affiliations:** Dipartimento di Chimica e Biologia, INSTM Research Unit, Università di Salerno, Via Giovanni Paolo II 132, 84084 Fisciano, Italy; bakunagendra@gmail.com (B.N.); evignola@unisa.it (E.V.); cdaniel@unisa.it (C.D.); prizzo@unisa.it (P.R.)

**Keywords:** planar orientation, nanoporous-crystalline films, 2D-WAXD, DSC

## Abstract

For poly(2,6-dimethyl-1,4-phenylene)oxide (PPO) films exhibiting nanoporous-crystalline (NC) phases, *c*_⊥_ orientation (i.e., crystalline polymer chain axes being preferentially perpendicular to the film plane) is obtained by crystallization of amorphous films, as induced by sorption of suitable low-molecular-mass guest molecules. The occurrence of *c*_⊥_ orientation is relevant for applications of NC PPO films because it markedly increases film transparency as well as guest diffusivity. Surprisingly, we show that the known crystallization procedures lead to *c*_⊥_ oriented thick (50–300 μm) films and to unoriented thin (≤20 μm) films. This absence of crystalline phase orientation for thin films is rationalized by fast guest sorption kinetics, which avoid co-crystallization in confined spaces and hence inhibit formation of flat-on lamellae. For thick films exhibiting *c*_⊥_ orientation, sigmoid kinetics of guest sorption and of thickening of PPO films are observed, with inflection points associated with guest-induced film plasticization. Corresponding crystallization kinetics are linear with time and show that co-crystal growth is poorly affected by film plasticization. An additional relevant result of this study is the linear relationship between WAXD crystallinity index and DSC melting enthalpy, which allows evaluation of melting enthalpy of the NC α form of PPO (ΔH_m_^ο^ = 42 ± 2 J/g).

## 1. Introduction

Orientation of polymer chain axes (*c* axes) in crystalline lamellae are perpendicular or nearly perpendicular (for monoclinic and triclinic unit cells) to the lamellar planes. Crystalline lamellae can be oriented with respect to the polymer film plane, mainly for films having a thickness lower than 1 μm. Common orientations are the so-called edge-on or flat-on [1,2,3,4,5,6,7], which lead to planar orientations of ***c*** axes being preferentially parallel or perpendicular to the film plane, respectively.

Even in the absence of mechanical stresses, different kinds of planar orientations [8,9,10,11,12,13,14] can be easily achieved for polymer films exhibiting co-crystalline (CC) phases, i.e., where a host polymer includes low-molecular-mass guest molecules in crystalline cavities [15,16,17,18,19,20,21]. In fact, even for thickness of several hundredths of microns, different kinds of planar and uniplanar orientations (i.e., preferential orientations of a crystalline axis or of a crystalline plane with respect to the film plane, respectively) have been observed for films exhibiting CC phases of syndiotactic polystyrene [15,16,17], poly-(L-lactic acid) [18,19] and poly(2,6-dimethyl-1,4-phenylene)oxide (PPO) [20,21]. 

The kind and degree of planar and uniplanar orientations are relevant for properties of CC polymer phases. In fact, in CC films, the orientation of the host polymer can induce orientation of active guest molecules, being fluorescent [22,23,24,25], magnetic [26,27], ferroelectric [28], photo-reactive [29] or chiral-optical [30].

planar and uniplanar orientations of CC phases are also maintained after guest removal procedures leading the nanoporous-crystalline (NC) phases: δ [31,32] and ε [33,34] for sPS, α and β for PPO [35,36,37]. This is mainly relevant for guest sorption kinetics because guest diffusivity can be largely changed by proper selection of the orientation of the NC phase [21,38,39,40,41]. Maximum diffusivity is generally observed for NC films with orientation of chain axes (and molecular channels) [17] preferentially perpendicular to the film plane [21,40,41]. This phenomenon is particularly relevant for NC PPO films with *c*_⊥_ orientation, which exhibit diffusivities comparable with those of NC powders and not far from those of NC aerogels [40]. As for PPO, it is also worth noting the much higher diffusivity of NC films with respect to amorphous films, although the latter are well known for their high free volume and fast diffusivities [42,43,44,45,46,47,48,49].

NC PPO films with *c*_⊥_ orientation also have the advantage of transparency comparable with those of amorphous PPO films [50]. This high transparency was rationalized on the basis of the positive birefringence of the polymer and of density and refractive index of the NC phases being lower than those of the corresponding amorphous phase [50]. Hence, PPO films with high guest diffusivity and transparency (both relevant for many possible applications) are achieved by using crystallization procedures leading to *c*_⊥_ orientation.

For PPO, *c*_⊥_ orientation was only obtained by slow guest induced crystallization in amorphous films [21,40,50] and was attributed to formation of flat-on crystalline lamellae in nanoconfined layers [51].

In the present paper, for films as obtained by guest (dibenzyl ether)-induced crystallization of amorphous films, we study the dependence of planar orientation on film thickness. The paper also compares kinetics of guest sorption with corresponding kinetics of crystallization and thickness variation of PPO amorphous films.

## 2. Materials and Methods

PPO with 350 kg/mol molecular weight (P6130 grade) was supplied by Sabic (Sittard, The Netherlands). Guest molecules: dibenzyl ether (BE) and chloroform (CHCl_3_) were purchased from Aldrich (Milan, Italy).

Amorphous PPO films with different thickness (~5–300 μm) were prepared by solution casting in chloroform under fast solvent evaporation conditions (atmospheric pressure and T ≈ 60 °C).

PPO CC films were prepared by guest-induced crystallization of amorphous films. Amorphous PPO films were immersed in liquid BE, at ~20 °C for immersion time in the range 5–2800 min. For kinetics studies, these CC PPO films just pulled out of BE liquid were immediately quenched in acetonitrile solvent in order to stop further crystallization.

PPO NC films were obtained from PPO CC films by guest extraction with supercritical carbon dioxide (scCO_2_) using SFX 200 equipment (ISCO Inc., Austin, TX, USA) at P = 250 bar, T = 40 °C and with extraction time t = 4 h. An alternative method of guest extraction, consisting of liquid acetonitrile sorption at room temperature followed by desorption in air again at room temperature, was also used.

Fourier transform infrared (FTIR) spectra were obtained at a 2.0 cm^−1^ resolution, with a Vertex 70 Bruker FTIR spectrometer (Bruker, Germany).

Differential Scanning Calorimetry (DSC) was conducted in TA Q2000 equipment from TA Instruments (New Castle, DE, USA). The reported DSC scans were conducted on NC PPO films at heating rate of 10 °C/min under controlled nitrogen gas flow.

Two-dimensional (2D) wide-angle X-ray diffraction (WAXD) patterns were performed in D8 QUEST Bruker diffractometer (CuK_α_ radiation, Bruker, Karlsruhe, Germany) with X-ray beam perpendicular and parallel to the film surface, thus obtaining patterns that are called THROUGH and EDGE, respectively.

Radial profiles were collected (by DIFFRAC.EVA software, Bruker, Karlsruhe, Germany) by averaging the intensities along circles, as a function of the diffraction angle (2θ). Equatorial and meridional profiles were collected along the equatorial and meridional directions of the collected 2D patterns.

Crystallinity indexes were determined by using radial profiles of 2D WAXD THROUGH patterns. In particular, we have applied the classical method of resolving these patterns into two areas corresponding to diffraction halo of the amorphous phase and to diffraction peaks of the crystalline phase, for 3° < 2θ < 30°.

The degree of orientation of the chain axes of the crystalline phase (*f_c_*) was evaluated by using the Herman’s orientation function:

(1) $$f_{c}=(3\bar{\cos^{2}\gamma }-1)/2$$

where cos^2^*γ* was determined by azimuthal scan of the 001 reflection of the NC α form, at 2θ_CuKα_ = 16.8°. In this framework, *f_c_* = 0 corresponds to unoriented crystalline phases, whereas *f_c_* = −0.5, corresponds to the limit *c*_⊥_ orientation, where chain axes of all crystallites are perpendicular to the film plane.

## 3. Results and Discussion

### 3.1. Dependence of Guest-Induced c_⊥_ Orientation on Film Thickness

**Figure 1:**
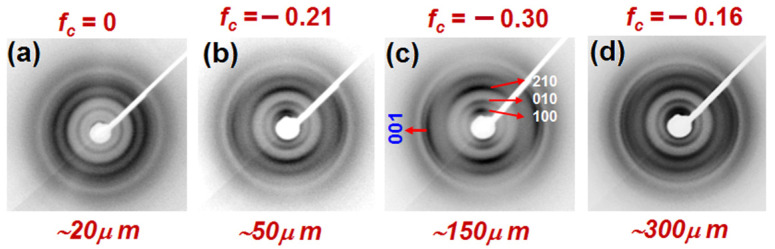
2D-WAXD EDGE patterns of NC PPO films, as obtained by BE induced crystallization in amorphous films with thickness of: (**a**) ~20 μm, (**b**) ~50 μm, (**c**) ~150 μm and (**d**) ~300 μm, after guest extraction. Degree of orientation (*f_c_*) is indicated at top of the patterns. The 001 reflection arc (centered on the equator) and the main hk0 reflection arcs (centered on the meridian), which show the occurrence of *c*_⊥_ orientation, are indicated in the pattern (**c**).

2D-WAXD patterns, as taken with the X-ray beam parallel to the film plane (EDGE patterns), of amorphous PPO films of different thickness in the range 20–300 μm, after crystallization induced by equilibrium room-temperature sorption of BE followed by BE desorption by scCO_2_, are shown in Figure 1. As discussed in [36], BE sorption leads to PPO/BE co-crystalline form, while the subsequent guest desorption leads to the NC α form.

For patterns of thicker films (from 50 μm up to 300 μm, Figure 1b–d), all hk0 reflections are present as arcs centered on the meridian. This clearly indicates, as already described for films of thickness of 50–90 μm [21,40,50], the occurrence of *c*_⊥_ orientation. Rather surprisingly, the thinner film (20 μm, Figure 1a) only exhibits diffraction rings, which clearly indicate absence of crystalline phase orientation.

**Figure 2:**
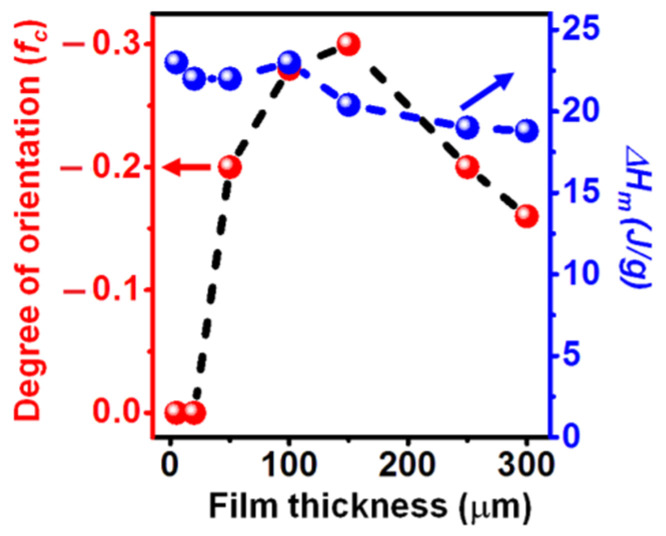
Degree of orientation (*f_c_*, left scale) and melting enthalpy (right scale) of NC PPO films versus thickness of the starting amorphous films. For *f_c_* = −0.5, all polymer chain axes of crystallites are perfectly perpendicular to the film plane.

The degree of orientation (*f_c_*, also indicated on the top of the patterns of Figure 1) is plotted versus thickness of seven starting amorphous PPO films, in Figure 2. For a better understanding of the plot of Figure 2, it is worth citing that the limit degree of *c*_⊥_ orientation, with all polymer chain axes of crystallites being perfectly perpendicular to the film plane, is *f_c_* = −0.5. Melting enthalpies of these films are also shown on the right scale in Figure 2. It is apparent that melting enthalpies and hence degrees of crystallinity are poorly dependent on film thickness while, on the contrary, the degree of crystalline phase orientation is maximum for the 150 μm film (*f_c_* = −0.3), it is substantially reduced for thicker films (e.g., *f_c_* = −0.16 for 300 μm) and it is negligible for thickness ≤ 20 μm.

Results strictly similar to those of Figure 1 and Figure 2 are observed if BE desorption occurs by sorption/desorption of acetonitrile, rather than by scCO_2_ extraction.

### 3.2. Kinetics of Be Guest Sorption from Amorphous Films of Different Thickness

This rather unexpected absence of crystalline phase orientation in thinner films can be rationalized in the framework of the recently proposed mechanism, for which *c*_⊥_ orientation is favored by slow uptakes of the guest-inducing polymer co-crystallization [51].

**Figure 3:**
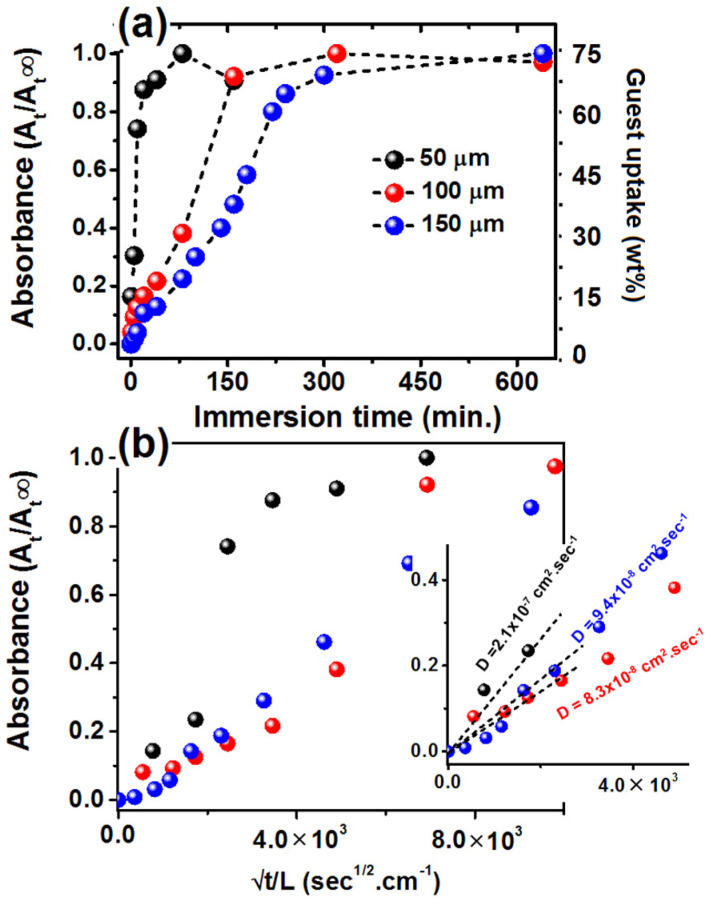
BE guest sorption kinetics at room temperature (~20 °C) in PPO amorphous films of different thickness (**a**) and corresponding Fick’s plots (**b**). The weight uptake for the 100 μm film is shown on the right scale. The inset in (**b**) shows enlarged Fick’s plot, which have been used to evaluate diffusivity values of BE in amorphous PPO films with different thickness.

BE sorption kinetics from amorphous PPO films of different thickness, based on the absorbance of the BE FTIR peak at 2215 cm^−1^, are shown in Figure 3. The kinetics of guest sorption of 5 μm and 20 μm film are not reported because they are too fast for the used FTIR method. The BE equilibrium uptake for all the PPO films is in the range 70–75 wt%, nearly independently of the thickness of the starting amorphous film, while it is reached in seconds, minutes and hours for 20 μm, 50 μm and 150 μm films, respectively. The average diffusivity for the kinetics of Figure 3a, as evaluated by the corresponding Fick’s plot up to variation of 30% (Figure 3b), is ~1.3 ± 0.5 × 10^−7^ cm^2^ sec^−1^.

The absence of orientation for guest-induced crystallization of thin films (Figure 2) is possibly associated with their fast guest sorption kinetics. In the framework of our hypothesis [51], fast guest sorption can avoid co-crystallization in confined spaces and hence formation of flat-on lamellae.

**Figure 4:**
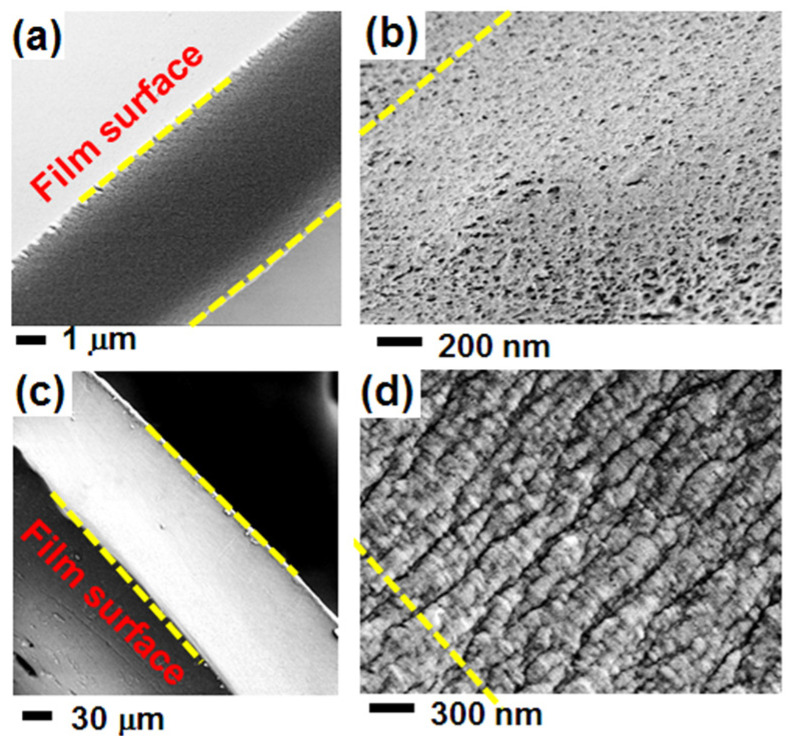
SEM images of sections of NC α form PPO films, as obtained by BE-induced crystallization on amorphous films, followed by BE extraction by scCO_2_. The thickness of the starting amorphous films is: (**a**,**b**) 20 μm; (**c**,**d**) 100 μm. The presence of flat-on lamellae is apparent only for the thicker film (**d**). The dashed yellow lines indicate the film surface direction.

This hypothesis is compatible with SEM images of sections of the NC films. For instance, SEM images of 20 μm and 100 μm amorphous films, after BE-induced crystallization followed by BE extraction by scCO_2_ are reported in Figure 4a–d, respectively. The presence of flat-on lamellae for the thicker film (Figure 4d) is apparent, as is the presence of an unoriented fibrillar morphology for the thinner film (Figure 4b).

### 3.3. Kinetics of Guest-Induced Film Thickening and Polymer Crystallization

Guest uptake in PPO amorphous films induces polymer co-crystallization and leads to remarkable increases (even doubling) of film thickness, which remains essentially unaltered even after complete guest removal).

**Figure 5:**
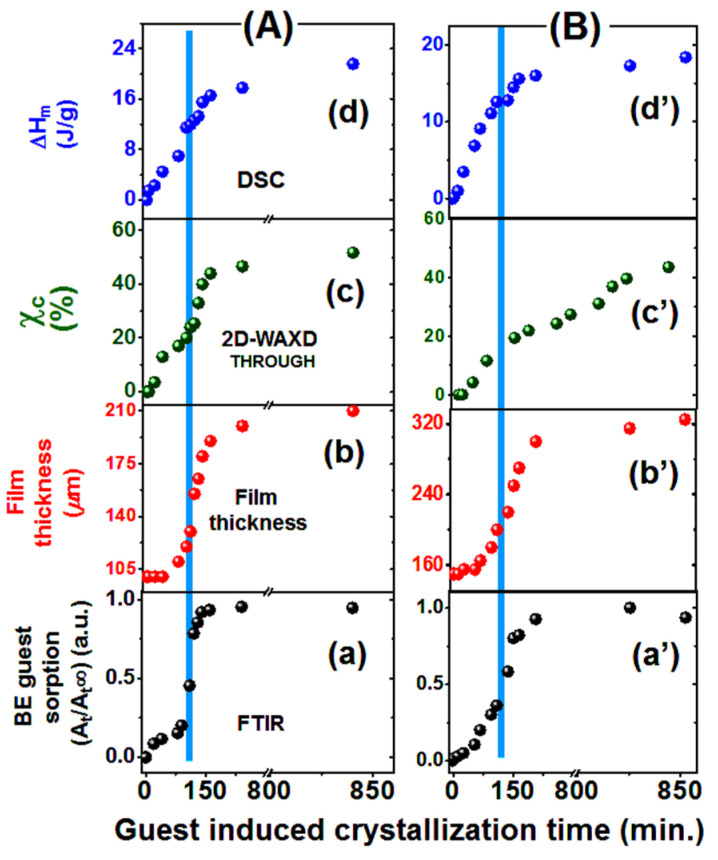
Kinetics of phenomena associated with room-temperature guest-induced crystallization of amorphous PPO films, with thickness of 100 μm (**A**) and 150 μm (**B**): (**a**,**a’**) BE guest sorption; (**b**,**b’**) film thickness; (**c**,**c’**) crystallinity index by WAXD (χ_c_); (**d**,**d’**) melting enthalpy (ΔH_m_). The blue vertical bar indicates the time of inflection of the S-shaped curve describing guest sorption, which can be taken as a measure of the time needed for film plasticization.

Kinetics of BE guest sorption (curves a and a’) and kinetics of film thickening (curves b and b’), for films with thickness of 100 μm and 150 μm, are compared in Figure 5A,B, respectively. It is apparent that guest uptake and thickness increase exhibit similar kinetics, with similar sigmoid shapes.

This sigmoid behavior is possibly related to the well-known progressive T_g_ reduction as a consequence of sorption of plasticizing molecules in amorphous phases [52]. When, for high guest content, T_g_ becomes close to room temperature, the transformation of the glassy phase in a rubbery phase leads to a jump in guest uptake as well as in film thickness.

Kinetics of crystallization as obtained by WAXD and DSC measurements on the NC films are shown as curves c,c’ and d,d’ in Figure 5A,B, respectively.

**Figure 6:**
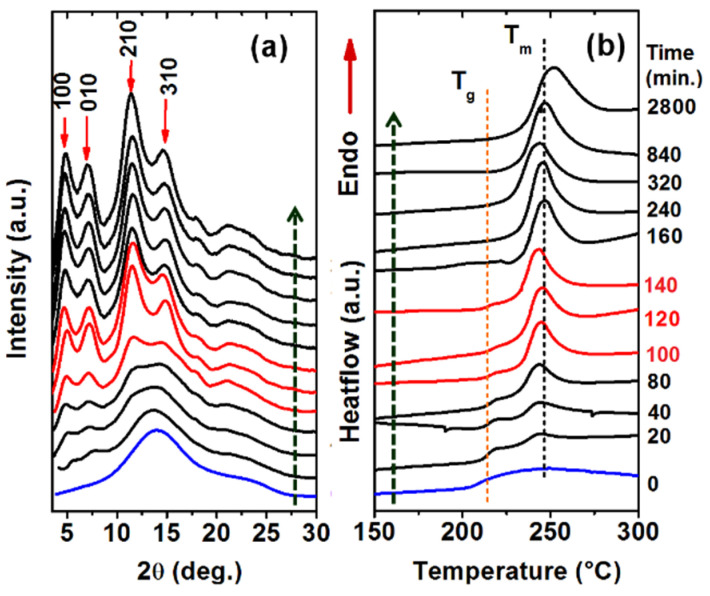
NC α form films, as obtained by sorption/desorption of BE in an amorphous 100 μm PPO film for different sorption times: (**a**) radial profiles of 2D-WAXD THROUGH patterns; (**b**) DSC scans at h.r. = 10 °C/min.

2D-WAXD patterns (as taken with the X-ray beam perpendicular to the film plane, THROUGH patterns) of amorphous PPO films, after crystallization induced by BE guest sorption/desorption, only exhibit diffraction rings [21]. Radial diffraction profiles of these 2D-WAXD THROUGH patterns, for a 100 μm film, after different sorption times (in the range 20–2800 min) are shown in Figure 6a. It is clearly apparent that, as a consequence of sorption, diffraction peaks typical of the NC α form (e.g., 100, 010, 210 and 310) [35,36] appear and progressively increase their intensity. Crystallinity indexes (χ_c_) as evaluated on the basis of the WAXD THROUGH patterns for 100 μm and 150 μm films are collected in the third column of Table 1 and Table 2 and plotted in curves c and c’ of Figure 5A,B, respectively.

DSC scans of the NC α form films of Figure 6a are shown in Figure 6b. For the amorphous as well as for the poorly crystallized films (corresponding to sorption time < 100 min), the PPO glass transition temperature (T_g_), at nearly 220 °C, is clearly apparent. As the sorption time increases, a melting peak appears, and its area progressively increases up to a melting enthalpy close to 22 J/g. Melting enthalpies as evaluated for films crystallized after different sorption times for 100 μm and 150 μm films are collected in the fourth column of Table 1 and Table 2 and plotted in curves d and d’ of Figure 5A,B, respectively. It is worth noting that for PPO samples, melting enthalpy is an appropriate quantity to evaluate degree of crystallinity, due to the negligible PPO re-crystallization during DSC heating scans [53,54].

**Figure 7:**
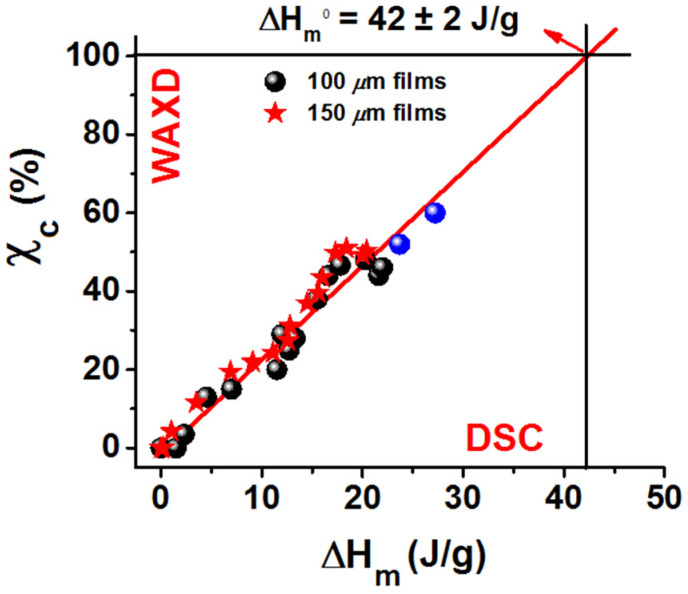
Crystallinity index (χ_c_, from WAXD patterns) and melting enthalpy (ΔH_m_, from DSC scans) of NC α form films, obtained by BE guest-induced crystallization of amorphous PPO films of different thickness: (●) 100 μm; (★) 150 μm. The blue circles indicate 100 μm films as crystallized after 2800 min immersion in BE and subsequently annealed at 40 °C and 60 °C. The red line indicated the extrapolation to a fully crystalline α-form PPO sample.

For both films with thickness of 100 μm and 150 μm, crystallinity indexes (χ_c_, as evaluated on the basis of the WAXD patterns, like those of Figure 6a) are plotted versus melting enthalpies (ΔH_m_, as evaluated by DSC scans, like those of Figure 6b) in Figure 7. A linear relationship between χ_c_ and ΔH_m_ appears, whose extrapolation to χ_c_ = 100% allows a rough evaluation of the melting enthalpy of fully crystalline α form PPO samples (ΔH_m_^ο^ = 42 ± 2 J/g). To reduce the extrapolation error, two additional points, corresponding to the 100 μm film after 2800 min of BE sorption followed by annealing at 40 °C and 60 °C for 12 h (last two rows of Table 1), are added. This ΔH_m_^ο^ value as obtained by the extrapolation procedure of Figure 7 compares well with the value as determined by measuring the depression of melting temperature in mixtures with chloronaphthalene (42 ± 8 J/g) [55].

Plots of Figure 5 show that crystallinity index (plots c) and melting enthalpy (plots d) present similar kinetics, with roughly linear increases before gradual approach to their plateau values, rather than sigmoid increases as observed for guest uptake (plots a) and thickness increase (plots b). This indicates that PPO crystallization, in the co-crystalline form with BE guest, occurs already for low guest concentrations in confined layers imposing formation of flat-on lamellae. By using the terminology of solvent-induced crystallization (often indicated as SINC) of amorphous films [56,57,58,59,60,61], the kinetics of crystallization is controlled by crystal growth rather than by guest diffusion. Kinetics of crystallization being linear with time indicate that co-crystal growth is poorly affected by film plasticization.

## 4. Conclusions

The planar orientation exhibiting crystalline chain axes preferentially perpendicular to the film plane (*c*_⊥_ orientation), which is observed for CC and NC PPO films as obtained by bulky guest (e.g., benzyl ether)-induced crystallization, depends on the thickness of the starting amorphous film. In fact, surprisingly, *c*_⊥_ orientation occurs only for thick films (50–300 μm), while it is not observed for films with thickness lower than or equal to 20 μm.

This unexpected behavior can be rationalized in the framework of the mechanism of planar oriented crystallization as proposed in a recent paper [51]. In fact, the absence of orientation for guest-induced crystallization of thin films is associated with fast guest sorption, which avoids co-crystallization in confined spaces and hence hinders formation of flat-on lamellae.

Kinetics of guest uptake and film thickening exhibit sigmoid shapes, which are due to the progressive T_g_ reduction as a consequence of sorption of plasticizing molecules in amorphous phases [61]. When for high guest content T_g_ becomes close to room temperature, the transformation of the glassy phase in a rubbery phase leads to a jump in guest uptake as well as in film thickness. 

Crystallization kinetics, as measured by WAXD and DSC techniques, show a completely different behavior with degree of crystallinity increasing linearly with sorption time before approaching their plateau values. This indicates that kinetics of PPO guest-induced co-crystallization, leading for thick films to *c*_⊥_ orientation, is controlled by crystal growth rather than by guest diffusion.

An additional relevant result of this study is the linear relationship between WAXD crystallinity index and DSC melting enthalpy, which allows for the evaluation of melting enthalpy of a 100% α form PPO sample (ΔH_m_^ο^ = 42 ± 2 J/g).

## Figures and Tables

**Table 1:** BE guest sorption at room temperature in amorphous PPO films, with thickness of ~100 μm, followed by guest removal leading to NC α form: sorption time (min); BE guest uptake (A_t_/A_t_∞); WAXD crystallinity index (%); melting enthalpy (J/g); film thickness (μm).

Sorption Time (min)	BE Guest Sorption (A_t_/A_t_∞)	Crystallinity Index WAXD (%) (±2)	Melting Enthalpy (ΔH_m_) in J/g (±0.5)	Film Thickness (μm) (±4)
0	0	0	0	100
5	0.08	0	1.5	100
20	0.11	3.5	2.3	100
40	0.15	13	4.5	100
80	0.20	15	7.0	110
100	0.45	20	11.5	120
110	0.78	29	12.0	130
120	0.85	25	12.7	155
130	0.91	28	13.3	165
140	0.93	38	15.5	180
160	0.94	44	16.6	190
240	0.95	46.6	17.8	200
320	1	48	20.3	200
840	0.94	44	21.6	210
2800	0.95	46	22.0	210
annealed at 40 °C	-	52	23.4	210
annealed at 60 °C	-	60	27.1	210

**Table 2:** BE guest sorption at room temperature in amorphous PPO films, with thickness of ~150 μm, followed by guest removal leading to NC α form: sorption time (min); BE guest uptake (A_t_/A_t_∞); WAXD crystallinity index (%); melting enthalpy (J/g); film thickness (μm).

Guest Sorption Time (min)	BE Guest Sorption (A_t_/A_t_∞)	Crystallinity Index WAXD (%) (±2)	Melting Enthalpy (ΔH_m_) in J/g (±0.5)	Film Thickness (μm) (±4)
0	0	0	0	150
5	0.01	0	0.2	150
20	0.10	4.2	1.02	150
40	0.12	11.6	3.5	155
80	0.22	19.4	6.9	155
100	0.30	21.9	9.1	165
140	0.42	24.3	11.1	180
160	0.48	27.4	12.6	200
200	0.58	31.1	12.8	220
220	0.80	36.9	14.5	250
240	0.86	39.6	15.6	270
300	0.92	43.5	16	300
640	1.0	49.8	17.3	315
840	0.83	51	18.4	325
1280	0.73	49.2	20	345
2800	0.73	50.3	20.4	330

## Data Availability

The data presented in this study are available on request from the corresponding author.
